# Directions of the bilateral Cochlear Implant in Brazil

**DOI:** 10.1590/S1808-86942012000100001

**Published:** 2015-10-20

**Authors:** Miguel Angelo Hyppolito, Ricardo Ferreira Bento

**Affiliations:** PhD. Professor of Otorhinolaryngology - Medical School of Ribeirão Preto – USP.; Full Professor of Otorhinolaryngology - Medical School of the University of São Paulo - USP.

The cochlear implant electronic device is the most effective sensorial prosthesis in medical history. Indications for cochlear implants have undergone evolutionary times, all based on technological development, improvements in the surgical techniques and the training and qualification of the interdisciplinary teams involved in the process of deployment. Thistechnology can help people with varying degrees of hearing loss at different times of sensory deprivation, although it works better when indicated early on, during the first year of life.

The indication of bilateral cochlear implantation has been a controversial issue and is a reality in European countries and in the USA. The British healthcare system indicates the simultaneous bilateral cochlear implants for all children with severe to profound bilateral hearing loss, for their studies have shown a high cost-benefit ratio for the health programs subsidized by the government, both individual and social. Children under 1 year of age with simultaneous bilateral cochlear implants behave like normal hearing listeners at 4 years of age, with better use of hearing in quiet and in noisy environments, better development in understanding and speech production, social behavior of normal-hearing children, as well as school achievement and school interaction as normal-hearing children without having to resort to lip reading and sign language.

The unilateral cochlear implant provides hearing, but limits the localization and discrimination of sounds in noisy environments, making it hard for the individual to follow the conversation.

The bilateral cochlear implant rapidly promotes a homogeneous and organized cortical activity measured by the compound auditory evoked potential (CAEP). Its stimulation promotes P1-wave latency and amplitude similar to normal-hearing children of the same age range, proving a symmetrical bilateral development of the central auditory pathways. Spoken language acquisition is faster within the first year after bilateral cochlear implant compared to the unilateral implant and children who receive the second cochlear implant closer to the first have better speech perception with the use of the second implant.

The relatives of patients using two cochlear implants reported improvement in their children's overall behavior, with better speech intelligibility, better school performance and better speech perception after the second implant.

Thus, the indication of bilateral cochlear implant is a reality and is being considered by regulatory agencies of private healthcare insurance companies; and in due time it will be indicated to patients from the public healthcare system. Age must be considered when one thinks aboutthe possibility of cochlear implants in children, for it may independently affect hearing development.

The best performance in terms of sound localization in bilateral cochlear implant users depends on the head-shadow, the “squelch” and the binaural summation effects. These effects are important for understanding speech in noisy environments, timely locating the sound reaching each implanted ear. The **head-shadow** effect occurs by head obstruction and the sound arriving at the stimulated ear, improving the signal-to-noise ratio (SNR). The **binaural summation** is the result of central auditory processing, and it shows the capacity the central nervous auditory system has to integrate and use information from both ears. The **Squelch effect** is the auditory system'sability to use the information from both ears when speech and noise are spatially separated.

In order to indicate bilateral cochlear implant, an important condition to consider is the critical period for binaural auditory development, which favors early simultaneous implant. The central auditory pathways' plasticity, soon after early bilateral implantation is favorable in children implanted bilaterally, and sequentially to a second implant performed in a time interval shorter than 1 year. The short time between sequential implants and simultaneous cochlear implantation in children should be considered favorable to rehabilitation with greater opportunity for speech and language development. In adults, simultaneous or sequential bilateral cochlear implantsenhancelistening comfort, to be able to listen through both ears, with the benefit of sound localization.

When considering bilateral cochlear implants in children two paramount issues are:the hearing loss duration before the first surgery, which interferes with the development of oral language and language skills, and the interval between surgeries, which affects the development of the binaural auditory processing.

The timing of the indication and age to undergo surgery should be thoroughly discussed with the cochlear implant program team, which will aim at early intervention and better overall hearing, seeking to reduce existing difficulties, and providing auditory benefits, pursuing respect and social inclusion.

International studies have shown that if money is not the problem, bilateral cochlear implant would benefit all children with profound bilateral sensorineural hearing loss, and both ears must meet the pediatric audiological criteria.

Contraindications for the bilateral cochlear implant are children weighing less than 6kg and lack of family support. In the case of adults the contraindication is related to the duration of sound deprivation without the use of individual hearing aids.

To wait and reserve a non-implanted ear to a new technology or future treatment should be considered and one must not forget that there is a critical period for cortical development. Therefore, waiting for a new technology can be harmful, because the cerebral cortex would not take advantage of this new technology or new treatment. Despite delays in incorporating these technologies to healthcare in Brazil, on July 28, 2011 the National Agency of Supplemental Health (ANS) through its Normative Resolution - RN No. 261 - requires that health insurance companies should add as of January, 2012 in their lists of procedures, the cochlear implants, facilitating the access of patients insured by health plans to this important benefit that is already a reality in the Brazilian Public Healthcare System (SUS), not requiring court orders for theunilateral or bilateral cochlear implant surgery. Now, patients enrolled in the SUS must wait for when the Federal Government will provide the technology bilaterally, which is already a reality in many countries.

